# Preoperative estimation of the pathological breast tumor size in architectural distortions: a comparison of DM, DBT, US, CEM, and MRI

**DOI:** 10.1007/s00330-025-11502-7

**Published:** 2025-03-20

**Authors:** Javier Azcona Sáenz, Javier Molero Calafell, Marta Román Expósito, Elisenda Vall Foraster, Laura Comerma Blesa, Rodrigo Alcántara Souza, María del Mar Vernet Tomás

**Affiliations:** 1 Radiology and Nuclear Medicine Department, Hospital del Mar, Barcelona, Spain; 2 Department of Medicine and Life Science (MELIS), Universitat Pompeu Fabra, Barcelona, Spain; 3 Department of Epidemiology and Evaluation, Hospital del Mar Medical Research Institute, Barcelona, Spain; 4 Pathology Department, Hospital del Mar, Barcelona, Spain; 5 Departament de Medicina, Universitat Autònoma de Barcelona, Barcelona, Spain; 6 Obstetrics and Gynaecology Department, Hospital del Mar, Barcelona, Spain

**Keywords:** Breast neoplasms, Neoplasm staging, Mammography, Ultrasonography, Magnetic resonance imaging

## Abstract

**Objective:**

This study aims to compare the accuracy of digital mammography (DM), digital breast tomosynthesis (DBT), ultrasound (US), magnetic resonance imaging (MRI), and contrast-enhanced mammography (CEM) in the preoperative evaluation of breast cancer size in architectural distortions (ADs). Additionally, it assesses whether including thin spicules in mammography measurements affects accuracy.

**Materials and methods:**

We planned a retrospective analysis of invasive breast cancers presenting as ADs in our breast screening program between 2018 and 2022. Tumor size was measured in mm using DM, DBT, US, MRI, and CEM. Measurements were compared to the surgical specimen sizes. Two measurement approaches for DM and DBT were applied, considering and not considering thin spicules. T-student test was used to compare mean sizes across imaging techniques with the surgical specimen.

**Results:**

The study encompassed 59 female patients with 63 ADs. Mean age was 60.1 years (Standard Deviation (SD): 6.3). The cancers included four histological subtypes, ductal (69.8%), lobular (23.8%), tubular (4.8%), and micropapillary (1.6%). All imaging techniques, except for US (mean: 12.4 mm, SD: 5.7), overestimated tumor size compared to histology (mean: 16.40 mm, SD: 9). CEM, MRI, and DBT without thin spicules closely matched histological size. Including thin spicules in DM and DBT led to overestimation. Concordance was highest with CEM (75%) and MRI (67.6%). No significant differences were found between ductal and lobular carcinoma.

**Conclusion:**

For preoperative tumor size estimation of breast cancer in ADs, DBT excluding thin spicules, CEM, and MRI seemed most accurate. Including thin spicules in mammography leads to overestimation.

**Key Points:**

***Question*** *Identifying the most accurate imaging technique for preoperative tumor staging of architectural distortions (ADs) is crucial now that contrast-enhanced mammography (CEM) is widely implemented.*

***Findings*** *Measuring thin wispy spicules in ADs on digital (DM) and digital breast tomosynthesis (DBT) should be avoided, as they consistently overestimate pathological tumor stage.*

***Clinical relevance*** *Precise tumor size estimation in ADs is critical for proper staging and treatment planning. This study favors the use of DBT excluding thin spicules, CEM, and magnetic resonance imaging (MRI) for optimal accuracy.*

**Graphical Abstract:**

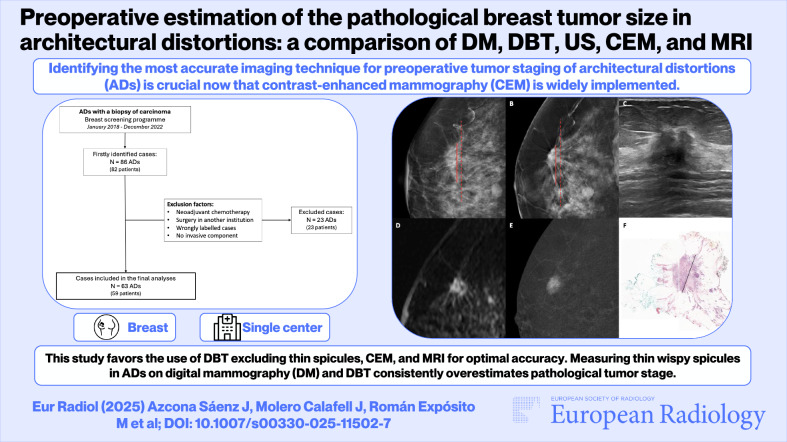

## Introduction

Architectural distortion (AD) is defined by the American College of Radiology (ACR) on the Breast Imaging Reporting and Data System (BI-RADS) as a distortion of normal breast architecture with no definite visible mass. For mammography, this includes spiculations radiating from a point, focal retraction, and straightening at the edges of the parenchyma. It can also be seen as an associated finding [1–4]. ADs represent nearly 6% of abnormalities detected on screening mammography [2] and can be caused by benign lesions such as post-surgical scars, radial scars, complex sclerosing lesions, sclerosing adenosis or fat necrosis, and malignant processes including invasive carcinoma or ductal carcinoma in situ [2, 3, 5–7]. ADs are less likely to represent malignancy on screening than on diagnostic mammography [4].

The maximum size of an AD can widely vary through different imaging techniques. The major discrepancy in measurements is typically seen on mammography depending on whether thin, wispy, spicules are included as part of the lesion.

On TNM staging, the T category of the primary tumor is defined by the maximum extension of the invasive component of the cancer [8] and is a well-known prognostic factor [9]. The pathological tumor (pT-) stage is preferably assessed by microscopic pathology [8], while the clinical tumor (cT-) stage is assessed by physical examination and imaging [9]. Many studies have confirmed the shortcomings of using physical examination alone to assess tumor size [10–15]. Therefore, cT-stage is normally assessed by imaging.

Because tumors are composed of varying proportions of noninvasive and invasive disease that imaging techniques are unable to distinguish [8] but pathology is, most clinicians and reports use pT-stage as the reference standard [9]. Nevertheless, some therapeutic decisions, such as preoperative chemotherapy, must be made based on cT-stage [9]. This issue prompts the question of how clinical and pathological tumor size correlate [9]. Underestimating cT-stage may lead to incomplete margins and, hence, re-excision [16, 17] while overestimating cT-stage may lead to non-indicated treatments and less conservative surgeries [18].

To our knowledge, there are no studies comparing the accuracy of DM, DBT, US, MRI, and CEM in cases of breast cancer that presents as an AD. Furthermore, we have only found three studies [17, 19, 20] that have compared the mammographic measurement of ADs including and excluding thin spicules to determine whether they should be measured.

The main purpose of this study was to evaluate for ADs which imaging technique correlates best with the surgical specimen invasive carcinoma size and to determine if mammographic thin spicules should be included in the measurement. Additionally, if the correlation between the imaging and the pathology tumor size varies depending on the histological subtype.

## Methods

### Study design

**Fig. 1:**
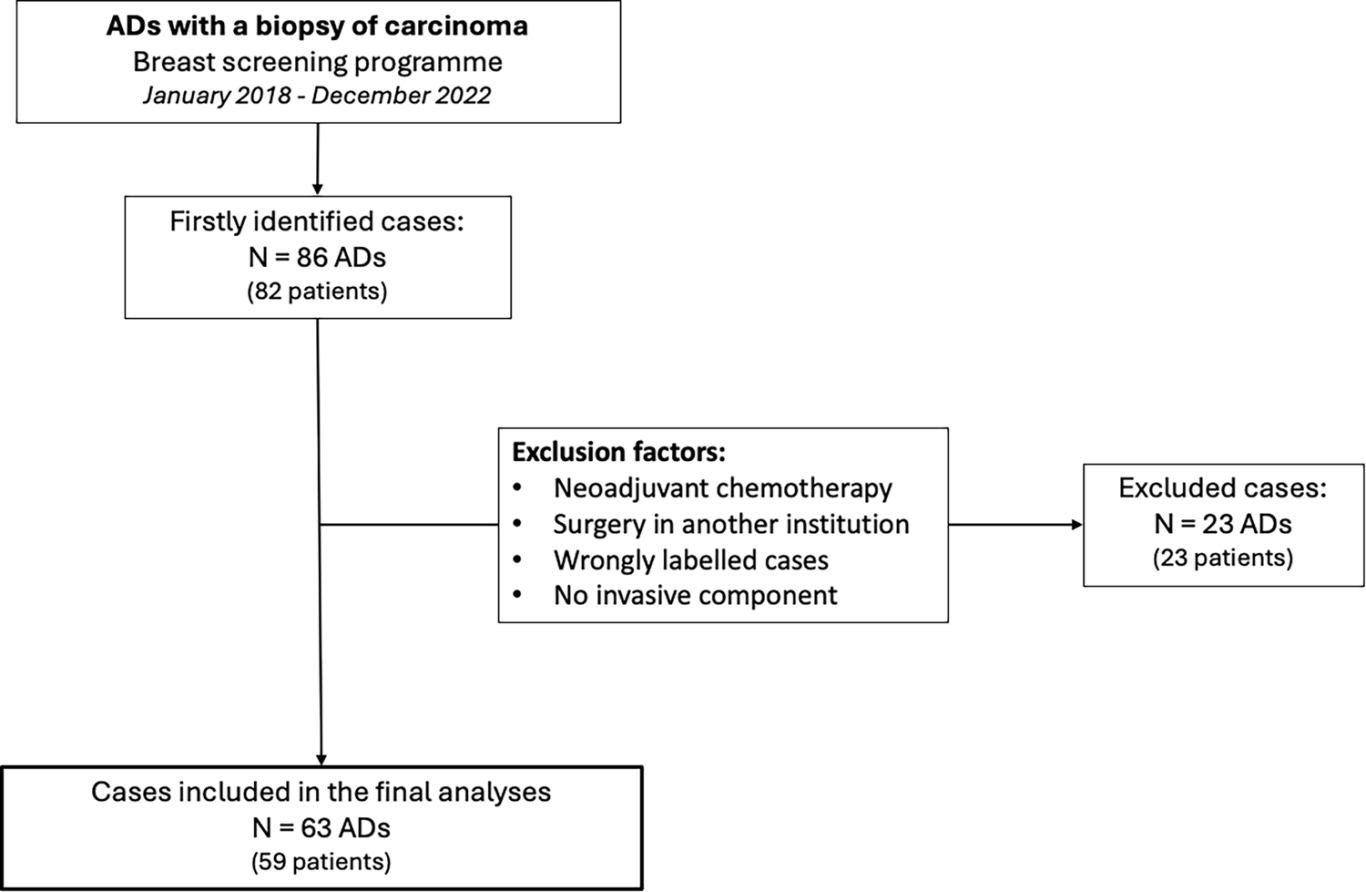
Flowchart of patient enrollment. From January 2018 to December 2022, all patients labeled as AD with a biopsy of carcinoma diagnosed at our breast screening programme were selected. Eighty-six cases of AD were obtained from 82 patients. After the exclusion factors, 63 cases of AD remained in 59 patients

We planned a retrospective cohort study. We reviewed the clinical records of patients labeled as AD and with a biopsy of carcinoma diagnosed at our breast screening programme between January 2018 and December 2022. Our screening programme consists of biannual DM performed on asymptomatic women aged 50 to 69 years. Given the retrospective nature of the study, the Ethical Committee waived the requirement for informed consent. Eighty-six cases of AD were identified from 82 patients. We excluded patients who underwent neoadjuvant chemotherapy, surgery in another institution, were wrongly labeled as AD, and those who presented with no invasive component. Finally, we gathered 63 ADs from 59 patients (Fig. 1).

### Imaging

The mammography equipment used was the Amulet Innovality system manufactured by Fujifilm Corporation and the Senographe Pristina system manufactured by GE Healthcare. Standard cranio-caudal and mediolateral-oblique views were employed for both DM and DBT, while additional lateromedial views were performed in cases of CEM. CEM entails the use of both high- and low-energy X-ray exposures, performed 2 min after intravenous iodinated contrast administration [21, 22]. For each view, three images are acquired: a low-energy, a high-energy, and a recombined image. However, only the low-energy image, which provides morphological information (equivalent to a standard DM) [23–25], and the recombined image derived by subtracting the low-energy image from the high-energy image, that assesses tumor neovascularity [24–26], are sent to PACS (picture archiving and communication system). The high-energy image is considered nondiagnostic. All DM were performed with Amulet Innovality, while DBT were performed with both Amulet Innovality and Pristina. All CEM were carried out with Pristina.

US examinations were performed with two ultrasound machines; LOGIC S7 EXPERT, and LOGIQ S8, both manufactured by GE Healthcare, using a 6–15 MHz probe. Images of the AD were obtained in at least the transversal and sagittal planes.

The MRI machine used was Signa Explorer 1.5 T, manufactured by General Electrics. A dedicated 7-channel phased-array coil was employed for image acquisition. Serial axial images were obtained through both breasts before and after the administration of gadolinium contrast, using compound imaging VIBRANT (volume imaging for breast assessment) T1-weighted sequencing. Computer analyses were done on GE Healthcare’s AW Server software.

The DM, DBT, US, MRI, and CEM images were retrieved for retrospective interpretation by just a single 2-year-experienced breast radiologist. The radiologist was informed about the study’s purpose but was blinded to the pT-stage, which is considered the gold standard.

All 63 lesions underwent DM as they were detected in our breast screening program. All recalled women with suspected AD underwent US, with 3 lesions (3/63, 4.8%) lacking ultrasound correlate. DBT, CEM, and MRI were performed based on the radiologist’s discretion, primarily for high-density breasts (ACR BI-RADS C and D), without clearly established criteria. Consequently, 29 lesions underwent DTB, 34 MRI and 40 CEM.

**Fig. 2:**
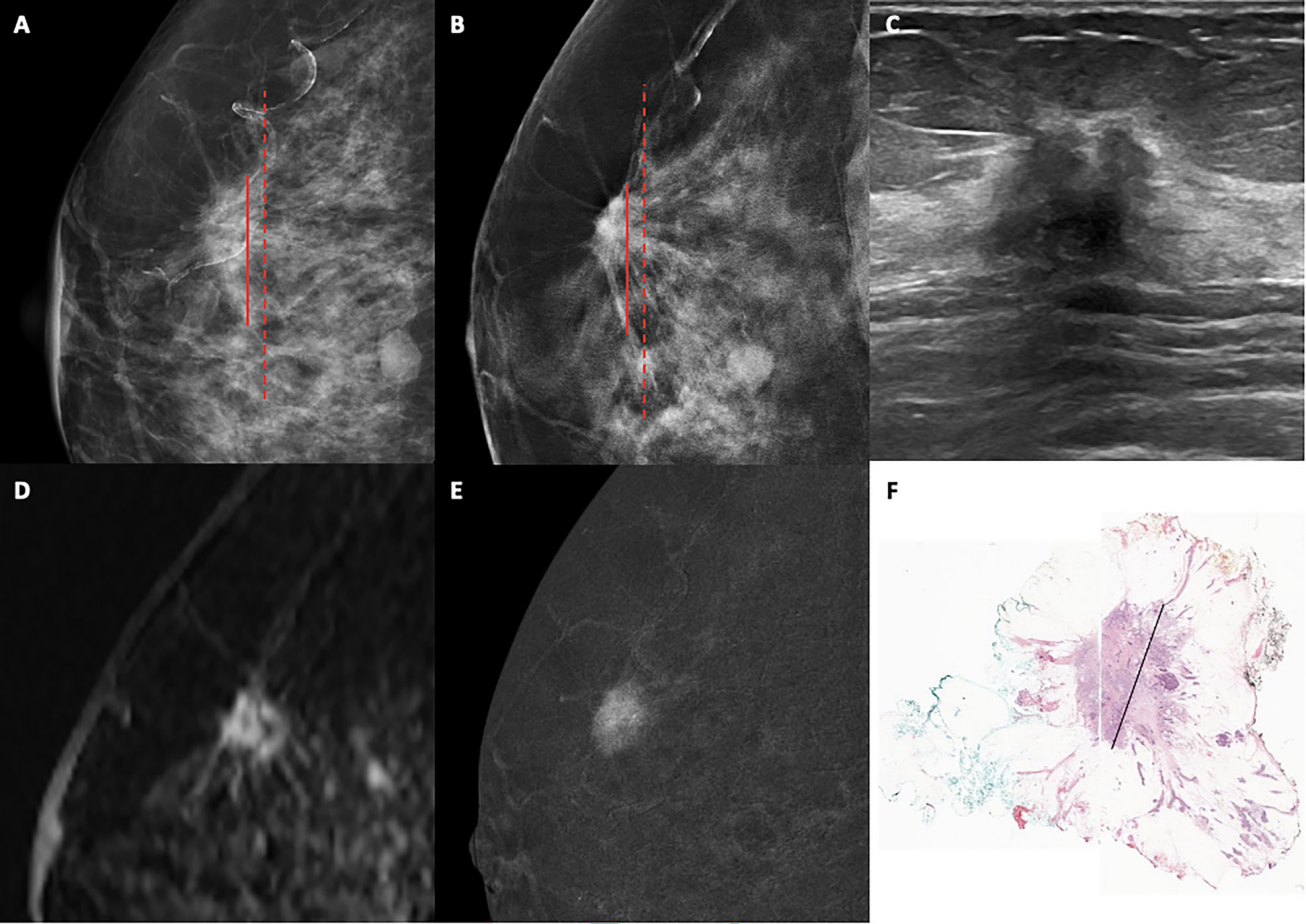
Architectural distortion found on a 51-year-old asymptomatic woman during breast cancer screening. **A** DM right cranio-caudal view. **B** DBT right cranio-caudal view. **C** Ultrasound transversal image. **D** MRI right sagittal subtraction image. **E** CEM (recombined image) right mediolateral view. **F** Hematoxylin and eosin tissue section of the lesion for microscopic evaluation. Red line on DM (**A**) and DBT (**B**) marks the maximum length of the AD’s core, including thick spicules but excluding thin, wispy, spicules. Red dashed line on DM (**A**) and DBT (**B**) marks the maximum length of the AD including thin, wispy, spicules. Black line on microscopic image (**F**) marks the largest diameter of the infiltrative lesion

For each lesion visible on DM, DBT, US, MRI and CEM, the maximum tumor size was measured to the nearest millimeter using GE Healthcare’s Universal PACS Viewer on 5-megapixel workstations Fig. 2. On DM and DBT two different measurements were performed, one of them considering only the core of the lesion, which includes thick spicules, and the other measurement, wider, including the maximum extension of the thin, wispy, spicules. On CEM, the maximum tumor size was only measured on the recombined images. On MRI, the maximum tumor length was measured on the first post-contrast series. As for the US examinations, the static US images of the lesions stored in PACS were reviewed and a measurement of the maximum tumor size was performed.

### Surgical specimen processing

All patients underwent surgical excision of the primary tumor. At the Pathology Department, each specimen was measured, and the margins inked with six colors for orientation, then sectioned into serial 4- to 5-mm-thick sections and photographed. Tumoral lesions were measured in three dimensions, and the distance to the surgical margins was recorded. After formalin fixation, the sections submitted for histological assessment were noted on the macroscopic photograph of the specimen. These sections were then submitted for paraffin embedding, sectioning, and staining with hematoxylin and eosin (HE) for histological evaluation. The maximum tumor size was measured to the nearest millimeter on the HE-stained tissue sections using either a metric eyepiece on a conventional microscope or a digital pathology software (uPath software, Roche) when digital pathology was available. Invasive tumor size was defined as the largest diameter of the invasive carcinoma.

### Study objectives

Our primary objective was to assess whether there were significant differences between the mean sizes obtained through each imaging technique and the mean size of all surgical specimens, regardless of the histological subtype. Additionally, the concordance between the measurements performed with each imaging modality and the pathological size was assessed.

As a secondary objective, we conducted an analysis to determine if there were significant differences between the mean measurements obtained from each imaging technique and the mean size of the two most common histological subtypes, ductal and lobular carcinoma.

### Statistical analysis

For the statistical analysis, the mean and standard deviation of lesion measurements were calculated. Paired *t*-tests (T-student) were performed to evaluate the mean difference between imaging measurements and pathology, under the null hypothesis of no difference between the means. All *t*-tests were two-sided, with significance defined as *p* < 0.05. Analyses were conducted using R version 4.3.2. We opted for the Student’s *t*-test instead of the Pearson’s correlation test, commonly used in other articles [9, 10, 19, 27–31]. The rationale behind this choice lies in the fact that a high correlation between two variables, even when measured on the same scale, does not necessarily imply equivalence. In certain cases, there might be a systematic deviation in one of the two measurements. Therefore, Pearson’s correlation test primarily measures association rather than agreement [16].

Scatter plots were designed to display the size in millimeters of each AD measured by the different imaging techniques in comparison to their size measured by microscopic pathology.

The measurements were considered concordant with histology if they were within ± 5 mm, underestimated if they were < 5 mm, and overestimated if they were > 5 mm, when compared to pathological size.

Further sensitivity analyses were conducted, including a complete case analysis of the five ADs examined by all imaging techniques (*n* = 5) and three subsets: DM-US-DBT-Histology (*n* = 29), DM-US-CEM-Histology (*n* = 40), and DM-US-MRI-Histology (*n* = 34).

For the mammographic classification on DM, we evaluated breast density using ACR BI-RADS categories [32]. Additionally, the histological subtypes of breast cancer were collected from the 63 ADs. T-student statistical test was performed to compare the mean on each imaging technique with the mean size of the two most common histological subtypes to analyze if the radio-pathological correlation of the AD varied depending on the histological subtype.

Since multiple statistical tests were performed, a correction for multiple comparisons was applied using the Holm-Bonferroni method, allowing us to obtain adjusted *p*-values (*p*_adj_).

## Results

Our study encompassed 59 female patients, with a mean age of 60.1 years (SD: 6.3). When evaluating breast density based on the ACR BI-RADS classification, 35 patients (59%) were classified as C, 20 (34%) as B, 2 (3.4%) as A, and 2 (3.4%) as D. Fifty-five patients presented with a single AD, while 4 patients presented with 2 ADs. The overall amount of ADs was 63.

Our analysis revealed no statistically significant variations (*p*_adj_ > 0.05) in the measurements obtained from CEM, MRI, DBT and DM (both excluding thin spicules) compared to the mean pT-stage of all surgical specimens. Conversely, we observed significant differences (*p*_adj_ < 0.05) in the mean sizes between US and both DM and DBT, including thin spicules, compared to the mean pT-stage of all surgical specimens (Table 1).

**Table 1:** Mean size and standard deviation (SD) of ADs on each imaging technique

	*N* = 63	Mean (SD)	*p*-value	Adjusted *p*-value
DM				
“With thin spicules”	63	33.9 (11)	*p* < 0.001	*p*_adj_ < 0.001
“Without thin spicules”	63	20.3 (8)	*p* < 0.05	*p*_adj_ > 0.05
DBT				
“With thin spicules”	29	35.5 (12)	*p* < 0.001	*p*_adj_ < 0.001
“Without thin spicules”	29	18.8 (9)	*p* > 0.05	*p*_adj_ > 0.05
Ultrasound	63	12.4 (5.7)	*p* < 0.01	*p*_adj_ < 0.05
CEM (recombined images)	40	19.0 (9)	*p* > 0.05	*p*_adj_ > 0.05
MRI	34	19.7 (9)	*p* > 0.05	*p*_adj_ > 0.05
Pathology	63	16.4 (9)		

T-student test’s *p*-value and adjusted *p*-value (*p*_adj_), using Holm-Bonferroni method, after comparing the mean of each imaging technique with the mean of all invasive carcinomas, regardless of the histological subtype

We did not find significant differences (*p*_adj_ > 0.05) between the mean of CEM, MRI, DBT and DM “without thin spicules” with the mean of all post-surgical invasive carcinomas. We did find significant differences (*p*_adj_ < 0.05) between the mean of DBT and DM “with thin spicules” and US with the mean of all post-surgical invasive carcinomas

**Fig. 3:**
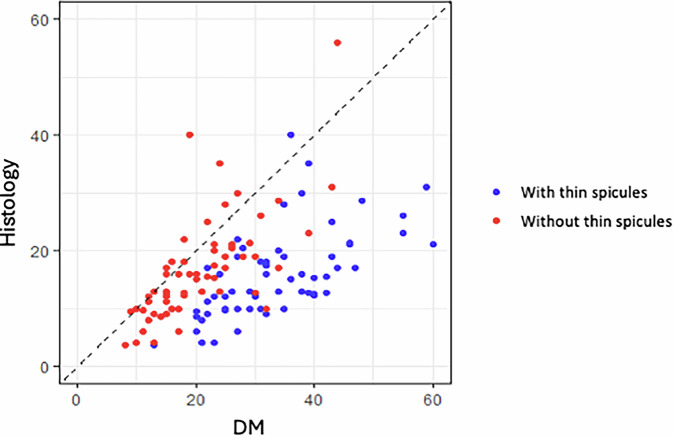
Scatter plot comparing DM “with thin spicules” and “without thin spicules” with post-surgical invasive carcinoma (Histology). Including the maximum extension of the thin spicules in the measurement of DM tends to clearly overestimate post-surgical invasive carcinoma. Thus, DM “without thin spicules” is more consistent with histology

**Fig. 4:**
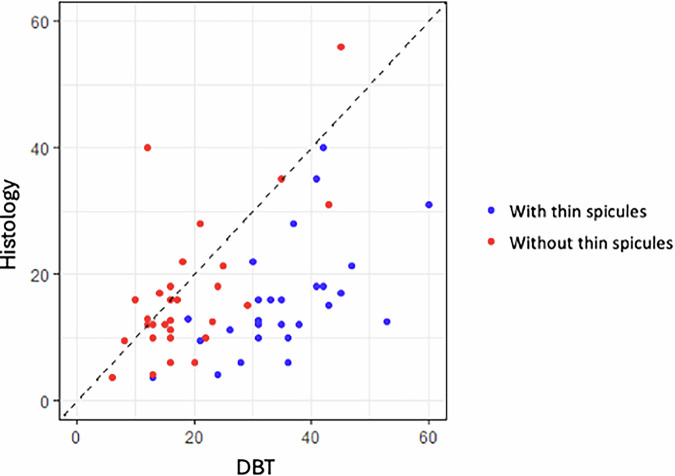
Scatter plot comparing DBT “with thin spicules” and “without thin spicules” with post-surgical invasive carcinoma (Histology). Including the maximum extension of the thin spicules in the measurement of DBT tends to clearly overestimate post-surgical invasive carcinoma. Thus, DBT “without thin spicules” is more consistent with histology

**Fig. 5:**
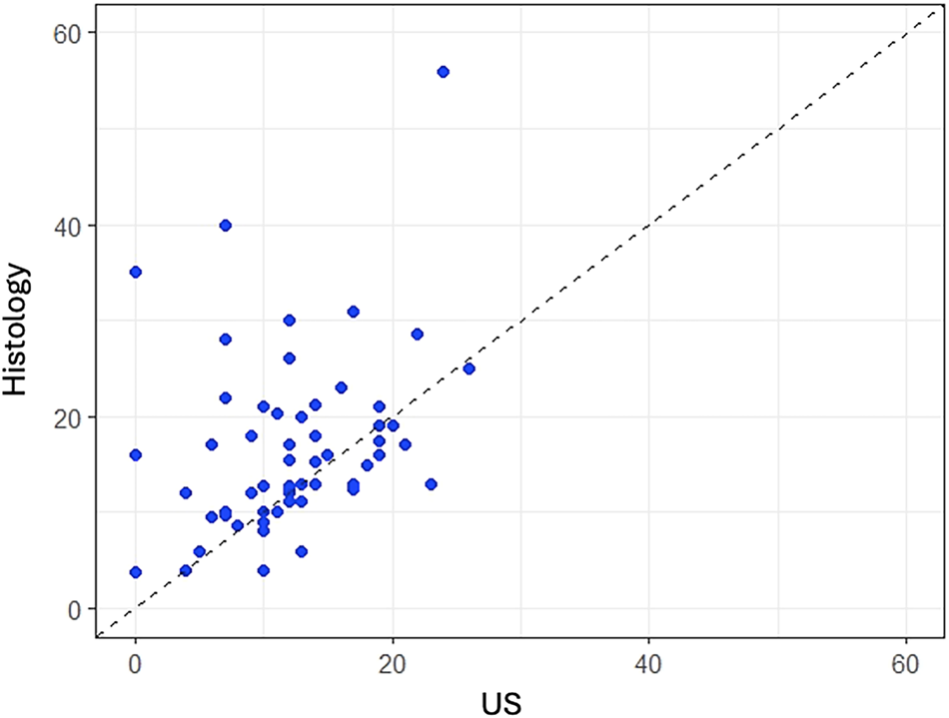
Scatter plot comparing US with post-surgical invasive carcinoma (Histology). Although data are quite scattered, in most cases US tends to underestimate invasive carcinoma

**Fig. 6:**
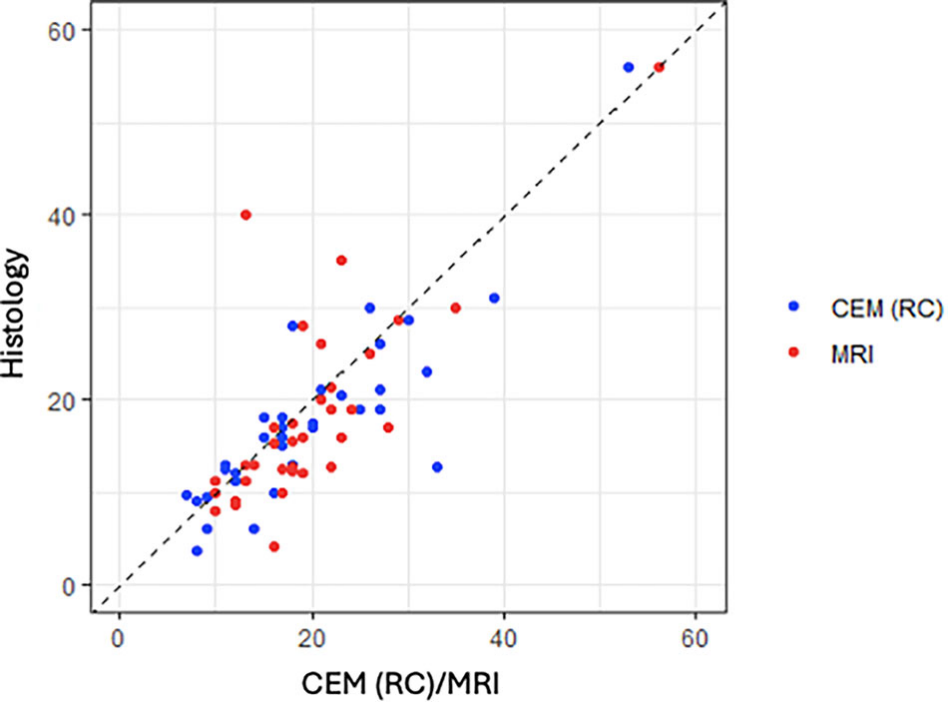
Scatter plot comparing CEM recombined images (RC) and MRI with post-surgical invasive carcinoma (Histology). CEM is more consistent with post-surgical invasive carcinoma than MRI. Both techniques tend to slightly overestimate post-invasive carcinoma

Scatter plots depicted some noteworthy findings. Including thin spicules in the mammographic measurements clearly overestimated invasive carcinoma (Figs. 3 and 4). Among the techniques, US exhibited the most scattered data and generally tended to underestimate invasive carcinoma (Fig. 5). While both MRI and CEM slightly overestimated invasive carcinoma, CEM was more consistent with pT-stage (Fig. 6).

Findings on the concordance of each imaging modality with the pathology size are summarized in Table 2. In the case of DM, including thin spicules in AD measurements led to an overestimation in 93.7% of cases. Conversely, excluding thin spicules from the measurements reduced the overestimation to 44.4% and improved the concordance. DBT exhibited patterns like those observed with DM. On the other hand, CEM, MRI, and US demonstrated the highest levels of concordance at 75%, 67.6%, and 65.1%, respectively. Notably, US had the highest rate of underestimation (25.4%).

**Table 2:** Concordance (± 5 mm) between imaging modalities and final histological examination

Concordance	DM				DBT				US		CEM		MRI	
	“With thin spicules”		“Without thin spicules”		“With thin spicules”		“Without thin spicules”							
	*N* (%)	Mean (SD)	*N* (%)	Mean (SD)	*N* (%)	Mean (SD)	*N* (%)	Mean (SD)	*N* (%)	Mean (SD)	*N* (%)	Mean (SD)	*N* (%)	Mean (SD)
Underestimated	-	-	3 (4.8%)	29.0 (13.2)	-	-	4 (13.8%)	22.0 (16.1)	16 (25.4%)	11.9 (5.7)	1 (2.5%)	18.0 (-)	3 (8.8%)	18.3 (5.0)
Concordant	4 (6.3%)	31.0 (7.8)	32 (50.7%)	16.7 (5.6)	2 (6.9%)	27.5 (20.5)	16 (55.2%)	15.8 (6.7)	41 (65.1%)	13.2 (4.8)	30 (75%)	17.0 (9.0)	23 (67.6%)	19.7 (10.2)
Overestimated	59 (93.7%)	34.1 (11.0)	28 (44.4%)	23.6 (8.2)	27 (93.1%)	36.1 (11.9)	9 (31%)	22.9 (9.0)	3 (4.8%)	15.3 (6.8)	9 (22.5%)	25.7 (8.4)	8 (23.5%)	20.1 (4.0)
Not visible	-	-	-	-	-	-	-	-	3 (4.8%)	0 (0)	-	-	-	-
Not available	-	-	-	-	34	-	34	-	-	-	23	-	29	-
Total	63													

Most concordant techniques are CEM and MRI. Measuring thin spicules on mammography overestimates invasive carcinoma in most cases. US is the imaging technique that more frequently underestimates invasive carcinoma

From a histological perspective, four different subtypes of breast cancer were exhibited. The most frequent subtype was ductal carcinoma (69.8%), followed by lobular carcinoma (23.8%). Tubular and micropapillary carcinoma only represented 4.8% and 1.6% of all cases, respectively. A comprehensive breakdown of breast cancer histological subtypes can be found in Table 3.

**Table 3:** Breast cancer histological subtypes of all ADs analyzed in our series

	*n*	%
Ductal carcinoma	44	69.8
IDC NST	10	15.9
IDC NST + DCIS	34	53.9
Lobular carcinoma	15	23.8
ILC	1	1.6
ILC + LCIS	14	22.2
Tubular carcinoma	3	4.8
Invasive tubular carcinoma	1	1.6
Invasive + in situ tubular carcinoma	2	3.2
Micropapillary carcinoma	1	1.6
Invasive + in situ micropapillary carcinoma	1	1.6
Total	63	100

Ductal carcinoma was the most common presentation (69.8%), followed by lobular carcinoma (23.8%), tubular carcinoma (4.8%) and micropapillary carcinoma (1.6%)

For invasive ductal carcinoma, our analysis revealed no significant differences (*p*_adj_ > 0.05) between the mean measurements obtained from DBT excluding thin spicules, US, CEM, and MRI when compared to the mean size of post-surgical invasive ductal carcinoma. However, we did observe significant differences (*p*_adj_ < 0.05) between the mean measurements obtained from DM including and excluding thin spicules, and DBT including thin spicules, when compared to the mean size of post-surgical invasive ductal carcinoma.

Concerning invasive lobular carcinoma, our analysis did not reveal significant differences between the mean measurements obtained from DM and DBT excluding thin spicules, DBT including thin spicules, US, CEM, and MRI, when compared to the mean size of post-surgical invasive lobular carcinoma. However, we did find significant differences (*p*_adj_ < 0.05) between the mean measurements obtained from DM including thin spicules in comparison to the mean size of post-surgical invasive lobular carcinoma. A detailed summary of these observations is presented in Table 4.

**Table 4:** Mean size and standard deviation (SD) of ADs on each imaging technique for ductal and lobular subtypes

	Ductal				Lobular			
	*N* = 44	Mean (SD)	*p*-value	Adjusted *p*-value	*N* = 15	Mean (SD)	*p*-value	Adjusted *p*-value
DM								
“With thin spicules”	44	32.8 (9.7)	*p* < 0.001	*p*_ajd_ < 0.001	15	37.5 (13.7)	*p* < 0.01	*p*_adj_ < 0.05
“Without thin spicules”	44	19.5 (6.7)	*p* < 0.001	*p*_adj_ < 0.01	15	23.7 (11.5)	*p* > 0.05	*p*_adj_ > 0.05
DBT								
“With thin spicules”	20	32.6 (9.1)	*p* < 0.001	*p*_adj_ < 0.001	8	43.4 (16.8)	*p* < 0.01	*p*_adj_ > 0.05
“Without thin spicules”	20	15.9 (5.0)	*p* > 0.05	*p*_adj_ > 0.05	8	26.1 (14)	*p* > 0.05	*p*_adj_ > 0.05
Ultrasound	44	12.4 (4.6)	*p* > 0.05	*p*_adj_ > 0.05	15	12.5 (7.7)	*p* < 0.05	*p*_adj_ > 0.05
CEM (recombined images)	28	17.4 (6.9)	*p* > 0.05	*p*_adj_ > 0.05	12	25.8 (12.7)	*p* > 0.05	*p*_adj_ > 0.05
MRI	22	16.7 (4.3)	*p* > 0.05	*p*_adj_ > 0.05	10	25.8 (12.7)	*p* > 0.05	*p*_adj_ > 0.05
Pathology	44	14.5 (6.3)			15	22.1 (13.0)		

T-student test’s *p*-value and adjusted *p*-value (*p*_adj_), using Holm-Bonferroni method, after comparing the mean of each imaging technique with the mean of ductal and lobular carcinomas

We did not find significant differences (*p*_adj_ > 0.05) between the mean of DBT “without thin spicules,” US, CEM and MRI and the mean of ductal and invasive carcinoma. In the case of DM “without thin spicules” there were no significant differences (*p*_adj_ > 0.05) for lobular carcinoma, but there were significant differences (*p*_adj_ < 0.05) for ductal carcinoma. Similarly, for DBT “with thin spicules” there were not significant differences (*p*_adj_ > 0.05) for lobular carcinoma, but significant differences (*p*_adj_ < 0.05) were identified for ductal carcinoma

Additional sensitivity analyses found in the supplementary material provided results that align with those presented in this manuscript.

## Discussion

To our knowledge, this is the first report that compares the accuracy of DM, DBT, US, MRI and CEM in cases of breast cancer that presents as an AD diagnosed at a breast screening program. In this series of 63 ADs diagnosed in 59 patients, the techniques that seemed most accurate for predicting pT-stage preoperatively, regardless of the histological subtype, were DBT excluding thin spicules, followed by CEM and MRI.

In our series, all imaging techniques tended to overestimate pT-stage, except for US, which underestimated it. Literature regarding US is contradictory; some articles are consistent with our results [16, 19, 28], while others state that US tends to overestimate pT-stage [33, 34]. Most literature reports state that DM underestimates spiculated lesions, due to difficulties in assessing their margins [19, 28]. MRI has been reported to slightly overestimate tumor size [18, 19, 29, 30, 33]. The most significant overestimations were observed in mammography measurements when thin spicules were considered. The closest estimates to the mean of pT-stage were from DBT excluding thin spicules, followed by CEM and MRI. Luparia et al described similar results regarding DBT and MRI, with a slightly higher correlation coefficient for MRI [19].

When assessing the concordance between imaging modality measurements and pathological size, morpho-functional techniques (CEM and MRI), exhibited the highest correlation values. Indeed, MRI typically offers the highest level of concordance with pathology [10, 19, 33, 35–37]. In recent years CEM has demonstrated excellent diagnostic performance [38–42] based on its high sensitivity, specificity, and low rate of false positives and negatives [21, 38]. Thus, CEM is nowadays considered a viable alternative for preoperative staging [18, 22, 38]. In fact, Fallenberg et al described a higher correlation between CEM and histopathology than with MRI [31]. In contrast, both DM and DBT demonstrated the least concordance when thin spicules were included within the measurements due to their high overestimation rates. Despite ranking third in concordance, US displayed the most considerable data dispersion and had the highest underestimation rate.

Regarding the distribution of pathology subtypes that we found in our cohort, our results are consistent with the ones reported by Bahl et al; they described similar proportions of invasive lobular carcinoma in their series, with approximately one-fourth of all invasive carcinomas identified as lobular [4].

When comparing the mean measurements of each imaging technique to the mean of the two most common histological subtypes, ductal and invasive carcinoma, the results were very similar. We did not find significant differences (*p*_adj_ > 0.05) between the mean of DBT excluding thin spicules, US, CEM and MRI when compared to the mean of ductal and lobular invasive carcinoma. In the case of DM, excluding thin spicules, there were no significant differences for lobular carcinoma, but significant differences (*p*_adj_ < 0.05) were observed for ductal carcinoma. Consequently, DM, excluding thin spicules, tends to overestimate lesion size in ductal carcinomas (+ 5 mm on average) more than in lobular carcinomas (+ 1.6 mm on average). This suggests that DM, excluding thin spicules, provides greater precision in measuring lobular carcinomas compared to ductal carcinomas—a finding that warrants investigation and may reflect the specific subset analyzed, as DM is classically less sensitive for invasive lobular carcinoma. Similarly, for DBT, including thin spicules, no significant differences (*p*_adj_ > 0.05) were observed for lobular carcinoma, but significant differences (*p*_adj_ < 0.05) were found for ductal carcinoma. In this case, measurements showed notable overestimation: + 18.1 mm for ductal carcinoma and + 21.3 mm for lobular carcinoma. In this instance, the adjusted *p*-value (0.055) slightly exceeded the threshold for significance, though the unadjusted *p*-value (< 0.01) suggests that DBT including spicules may be less precise for lobular carcinoma compared to DM, DBT excluding thin spicules, CEM, and MRI.

Most studies about the preoperative estimation of the pathological breast tumor size in ADs have performed the measurements only including the core of the lesion [9, 16, 28]. We have found just one article that performed all measurements including thin spicules [43].

The measurement of the maximum extension of thin spicules on mammography resulted in the most significant overestimation of invasive carcinoma and showed poor concordance when compared to pT-stage. This finding is consistent with previous studies conducted by Flanagan et al [17], Luparia et al [19] and Saif et al [20]. Thin spicules, in most cases, represent a fibrous stromal response to a tumor [17, 19, 28]. Importantly, these spicules do not significantly influence the pT-stage assessment. Hence, in the context of mammography, the most accurate correlation between radiological and pathological findings is achieved when thin spicules are disregarded.

Our study had several limitations. The study was conducted by just a single breast radiologist with 2 years of experience, which prevented the assessment of inter-observer agreement. Mean values and concordance between imaging modalities were assessed excluding not available cases which could bias the obtained results. The retrospective nature of the imaging review and the use of non-anonymized images could impact measurements across techniques. The chosen slicing plane in pathological assessments might not always capture the full tumor’s extent. Moreover, the small sample size should be considered. Future larger, prospective studies, free from limitations, are necessary to obtain consistent results.

In conclusion, the techniques that seem most suitable for assessing breast tumor size diagnosed through AD biopsy are DBT excluding thin spicules within the measurements, CEM, and MRI. DBT, excluding thin spicules, is promising as a preoperative noninvasive technique, especially for patients who cannot undergo CEM or MRI. These imaging techniques could serve as a viable alternative to stage the tumor when a precise pathological tumor size assessment is unattainable.

## Supplementary information

ELECTRONIC SUPPLEMENTARY MATERIAL

## Abbreviations

ACR: American College of Radiology
AD/ADs: Architectural distortion/architectural distortions
BI-RADS: Breast Imaging Reporting and Data System
CEM: Contrast-enhanced mammography
cT- stage: Clinical tumor stage
DBT: Digital breast tomosynthesis
DM: Digital mammography
HE: Hematoxylin and eosin
MRI: Magnetic resonance imaging
NST: No special type
PACS: Picture Archiving and Communication System
p_adj_: Adjusted *p*-value
pT- stage: Pathological tumor stage
SD: Standard deviation
US: Ultrasound
