# Supplementary material for: Preoperative estimation of the pathological breast tumor size in architectural distortions: a comparison of DM, DBT, US, CEM, and MRI
Source: Eur Radiol. 2025 Mar 20;35(9):5635–45. doi: 10.1007/s00330-025-11502-7 (PMC12350446; doi:10.1007/s00330-025-11502-7)
Supplement: Supplementary file 1 — ELECTRONIC SUPPLEMENTARY MATERIAL [file 330_2025_11502_MOESM1_ESM.pdf]

Preoperative estimation of the pathological breast tumor size in architectural distortions: A comparison of DM, DBT, US, CEM, and MRI

ELECTRONIC SUPPLEMENTARY MATERIAL

Complete case analysis of the sample (N = 5)

Supplementary Table 1. Mean tumor size (mm) and standard deviation (SD) for ADs on each imaging technique. T-student test's p-value and adjusted p-value (p_{adj}), using Holm-Bonferroni method, represent the comparison between the mean tumor size for each imaging technique and the mean size of all post-surgical invasive carcinomas, regardless of the histological subtype.

	N = 5	Mean (SD)	p-value	Adjusted p-value
**DM**				
"with thin spicules"	5	41.6 (13.7)	p>0.05	p_{adj}>0.05
"without thin spicules"	5	25.4 (11.3)	p>0.05	p_{adj}>0.05
**DBT**				
"with thin spicules"	5	44.2 (17.9)	p>0.05	p_{adj}>0.05
"without thin spicules"	5	23.0 (13.1)	p>0.05	p_{adj}>0.05
**Ultrasound**	5	16.6 (7.2)	p>0.05	p_{adj}>0.05
**CEM (recombined images)**	5	23.2 (17.0)	p>0.05	p_{adj}>0.05
**MRI**	5	24.4 (17.8)	p>0.05	p_{adj}>0.05
**Pathology**	5	25.3 (18.3)		

We did not find significant differences (p_{adj}>0.05) between the mean values of all imaging techniques (DM, DBT, US, CEM, and MRI) and the mean of all post-surgical invasive carcinomas, likely due to the small sample size (n=5). However, the imaging means closest to the pathology mean (25.3) were DM "without thin spicules" (25.4), MRI (24.4), CEM (23.2), and DBT without thin spicules (23.0). In contrast, the most divergent means were those of DBT (44.2) and DM (41.6) when thin spicules were included within the measurements.

Supplementary Table 2. Concordance (+/- 5 mm) between imaging modalities and final histological examination. This table presents the concordance rates between imaging techniques and final histology, categorized as underestimated, concordant, or overestimated.

	DM		DBT				
**Concordance**	**"with thin spicules"**	**"without thin spicules"**	**"with thin spicules"**	**"without thin spicules"**	**US**	**CEM**	**MRI**
**Underestimated**	-	1 (20%)	-	2 (40%)	2 (40%)	1 (20%)	1 (20%)
**Concordant**	-	2 (40%)	-	2 (40%)	2 (40%)	4 (80%)	4 (80%)
**Overestimated**	5 (100%)	2 (40%)	5 (100%)	1 (20%)	1 (20%)	-	-
**Total**	5 (100%)						

Most concordant techniques are CEM and MRI. Measuring thin spicules on mammography (DM and DBT) clearly overestimates invasive carcinoma, while ultrasound (US) underestimates it in 40% of cases.

Complete case analysis of three subsets of the sample: DM vs US vs DBT vs Histology (N = 29), DM vs US vs CEM vs Histology (N = 40), DM vs US vs MRI vs Histology (N = 34)

Supplementary Table 3. Mean tumor size (mm) and standard deviation (SD) for ADs measured using different imaging techniques in three subsets of the sample. T-student test's p-value and adjusted p-value (p_{adj}), using Holm-Bonferroni method, represent the comparison between the mean tumor size for each imaging technique and the mean size of all post-surgical invasive carcinomas in each subset, regardless of the histological subtype.

**Subset 1**	**N = 29**	**Mean (SD)**	**p-value**	**Adjusted p-value**
**DM**				
"with thin spicules"	29	31.8 (11.0)	$p<0.001$	$p_{adj}<0.001$
"without thin spicules"	29	19.4 (8.6)	$p>0.05$	$p_{adj}>0.05$
**Ultrasound**	29	10.8 (6.2)	$p<0.05$	$p_{adj}<0.05$
**DBT**				
"with thin spicules"	29	35.5 (12.3)	$p<0.001$	$p_{adj}<0.001$
"without thin spicules"	29	18.8 (9.3)	$p>0.05$	$p_{adj}>0.05$
**Pathology**	29	17.0 (11.5)		

**Subset 2**	**N = 40**	**Mean (SD)**	**p-value**	**Adjusted p-value**
**DM**				
"with thin spicules"	40	34.9 (12.0)	$p<0.001$	$p_{adj}<0.001$
"without thin spicules"	40	20.9 (8.8)	$p<0.05$	$p_{adj}>0.05$
**Ultrasound**	40	13.0 (5.6)	$p<0.05$	$p_{adj}>0.05$
**CEM (recombined images)**	40	19.0 (9.4)	$p>0.05$	$p_{adj}>0.05$
**Pathology**	40	16.8 (9.2)		

**Subset 3**	**N = 34**	**Mean (SD)**	**p-value**	**Adjusted p-value**
**DM**				
"with thin spicules"	34	36.0 (10.2)	$p<0.001$	$p_{adj}<0.001$
"without thin spicules"	34	22.1 (7.9)	$p>0.05$	$p_{adj}>0.05$
**Ultrasound**	34	13.6 (5.9)	$p<0.05$	$p_{adj}>0.05$
**MRI**	34	19.7 (8.6)	$p>0.05$	$p_{adj}>0.05$
**Pathology**	34	18.3 (10.5)		

We did not find significant differences ($p_{adj}>0.05$) between the mean of DBT and DM "without thin spicules", CEM, and MRI with the mean of post-surgical invasive carcinomas in all subsets.

We did find significant differences ($p_{adj}<0.05$) between the mean of DBT and DM “with thin spicules” with the mean of post-surgical invasive carcinomas in all subsets.

In the cases of Ultrasound, in subsets 2 and 3, we did not find significant differences ($p_{adj}>0.05$), while in subset 1 we did find significant differences ($p_{adj}<0.05$).

The fact that the sample size in each subset is larger than in **Supplementary Table 1** adds more value to the significance of each imaging test.

Furthermore, for each subset:

- In subset 1, the imaging mean closest to the histology mean was DBT “without thin spicules”, followed by DM “without thin spicules”.
- In subset 2, the imaging mean closest to the histology mean was CEM.
- In subset 3, the imaging mean closest to the histology mean was MRI, followed by DM “without thin spicules”.

In all three subsets, the mean from DM “with thin spicules” was the furthest from the histology mean.

Supplementary Table 4. Concordance (+/- 5 mm) between imaging modalities and final histological examination for each subset. This table presents the concordance rates between imaging techniques and final histology, categorized as underestimated, concordant, overestimated or not visible.

Subset 1	DM		US	DBT	
Concordance	"with thin spicules"	"without thin spicules"		"with thin spicules"	"without thin spicules"
Underestimated	-	3 (10.3%)	8 (27.6%)	-	4 (13.8%)
Concordant	3 (10.3%)	16 (55.2%)	15 (51.7%)	2 (6.9%)	16 (55.2%)
Overestimated	26 (89.7%)	10 (34.5%)	3 (10.3%)	27 (93.1%)	9 (31.0%)
Not visible	-	-	3 (10.3%)	-	-
Total	29				

In **subset 1**, DM and DBT "without thin spicules" were the most concordant imaging techniques (55.2%). However, levels of concordance among all three imaging techniques were very similar. Additionally, US had the highest tendency to underestimate histology (27.6%).

Subset 2	DM		US	CEM
Concordance	"with thin spicules"	"without thin spicules"		
Underestimated	-	1 (2.5%)	11 (27.5%)	1 (2.5%)
Concordant	1 (2.5%)	22 (55.0%)	25 (62.5%)	30 (75.0%)
Overestimated	39 (97.5%)	17 (42.5%)	2 (5.0%)	9 (22.5%)
Not visible	-	-	2 (5.0%)	-
Total	40			

In **subset 2**, CEM was the most concordant imaging technique (75.0%), followed by US (62.5%).

Subset 3	DM		US	MRI
Concordance	"with thin spicules"	"without thin spicules"		
Underestimated	-	3 (8.8%)	9 (26.5%)	3 (8.8%)

**Concordant**	2 (5.9%)	15 (44.1%)	22 (64.7%)	23 (67.7%)
**Overestimated**	32 (94.1%)	16 (47.1%)	2 (5.9%)	8 (23.5%)
**Not visible**	-	-	1 (2.9%)	-
**Total**	34			

In **subset 3**, MRI was the most concordant imaging technique (67.7%), followed by US (64.7%).

Supplementary Table 5. Mean size (mm) and standard deviation (SD) for ductal and lobular subtypes of ADs on each imaging technique for each subset.

T-student test's p-value and adjusted p-value (p_{adj}), using Holm-Bonferroni method, represent the comparison between the mean tumor size for each imaging technique and the mean size of ductal and lobular carcinomas in each subset.

Subset 1	Ductal				Lobular			
	N = 20	Mean (SD)	p-value	Adjusted p-value	N = 8	Mean (SD)	p-value	Adjusted p-value
**DM**								
"with thin spicules"	20	29.5 (7.4)	$p<0.001$	$p_{adj}<0.001$	8	38.1 (16.6)	$p>0.05$	$p_{adj}>0.05$
"without thin spicules"	20	17.8 (5.7)	$p>0.05$	$p_{adj}>0.05$	8	23.8 (13.4)	$p>0.05$	$p_{adj}>0.05$
**DBT**								
"with thin spicules"	20	32.6 (9.1)	$p<0.001$	$p_{adj}<0.001$	8	43.4 (16.8)	$p<0.05$	$p_{adj}>0.05$
"without thin spicules"	20	16.0 (5.0)	$p>0.05$	$p_{adj}>0.05$	8	26.1 (14.0)	$p>0.05$	$p_{adj}>0.05$
**Ultrasound**	20	11.2 (5.2)	$p>0.05$	$p_{adj}>0.05$	8	10.4 (8.9)	$p>0.05$	$p_{adj}>0.05$
**Pathology**	20	13.8 (7.5)			8	24.5 (16.6)		

Subset 2	Ductal				Lobular			
	N = 28	Mean (SD)	p-value	Adjusted p-value	N = 12	Mean (SD)	p-value	Adjusted p-value
**DM**								
"with thin spicules"	28	33.7 (10.7)	$p<0.001$	$p_{adj}<0.001$	12	37.7 (14.7)	$p<0.05$	$p_{adj}>0.05$
"without thin spicules"	28	19.7 (6.9)	$p<0.01$	$p_{adj}<0.01$	12	23.8 (12.1)	$p>0.05$	$p_{adj}>0.05$
**Ultrasound**	28	12.7 (5.0)	$p>0.05$	$p_{adj}>0.05$	12	13.6 (6.9)	$p>0.05$	$p_{adj}>0.05$
**CEM** (recombined images)	28	17.4 (6.9)	$p>0.05$	$p_{adj}>0.05$	12	22.7 (13.1)	$p>0.05$	$p_{adj}>0.05$
**Pathology**	28	14.4 (5.1)			12	22.3 (13.7)		

Subset 3	Ductal				Lobular			
	N = 22	Mean (SD)	p-value	Adjusted p-value	N = 10	Mean (SD)	p-value	Adjusted p-value
**DM**								
"with thin spicules"	22	33.7 (9.4)	$p<0.001$	$p_{adj}<0.001$	10	40.0 (11.7)	$p<0.05$	$p_{adj}>0.05$
"without thin spicules"	22	20.6 (6.4)	$p<0.05$	$p_{adj}<0.05$	10	26.0 (10.2)	$p>0.05$	$p_{adj}>0.05$
**Ultrasound**	22	13.1 (4.3)	$p>0.05$	$p_{adj}>0.05$	10	13.8 (8.2)	$p<0.05$	$p_{adj}>0.05$
**MRI**	22	16.7 (4.3)	$p>0.05$	$p_{adj}>0.05$	10	25.8 (12.7)	$p>0.05$	$p_{adj}>0.05$

**Pathology**	22	15.2 (7.5)			10	24.9 (13.9)		

We did not find significant differences (p_{adj}>0.05) between the mean values of DBT “without thin spicules”, US, CEM, and MRI and the mean of post-surgical invasive ductal and lobular carcinomas in all subsets.

In the case of DM “with thin spicules”, we did find significant differences (p_{adj}<0.05) for post-surgical invasive ductal carcinoma, but we did not find significant differences (p_{adj}>0.05) for post-surgical invasive lobular carcinoma.

In the case of DM “without thin spicules” we did not find significant differences (p_{adj}>0.05) for post-surgical invasive lobular carcinoma. However, we did find significant differences (p_{adj}<0.05) for post-surgical invasive ductal carcinoma, except in subset 1.
